# Reduced canine BRCA2 expression levels in mammary gland tumors

**DOI:** 10.1186/s12917-015-0483-9

**Published:** 2015-07-23

**Authors:** Yasunaga Yoshikawa, Masami Morimatsu, Kazuhiko Ochiai, Toshina Ishiguro-Oonuma, Seiichi Wada, Koichi Orino, Kiyotaka Watanabe

**Affiliations:** Laboratory of Veterinary Biochemistry, School of Veterinary Medicine, Kitasato University, Aomori, 034-8628 Japan; Laboratory of Laboratory Animal Science and Medicine, Department of Disease Control, Graduate School of Veterinary Medicine, Hokkaido University, Sapporo, 060-0818 Japan; Department of Basic Science, School of Veterinary Nursing and Technology, Nippon Veterinary and Life Science University, Tokyo, 180-8602 Japan; Department of Biological Resources, Integrated Center for Science, Ehime University, Ehime, 791-0295 Japan; Laboratory of Veterinary Radiology and Radiation Biology, School of Veterinary Medicine, Kitasato University, Aomori, 034-8628 Japan

**Keywords:** Splice variant, Nonsense-mediated mRNA decay, Premature termination codon, *BRCA2* promoter, Promoter variation

## Abstract

**Background:**

Mammary tumors are the most common tumor type in intact female dogs. Recently, the breast cancer 2 early onset (*BRCA2*) gene was proposed to be associated with tumorigenesis in dogs. The expression level of BRCA2 is important for its DNA repair function in mammalian cells, and its expression level is linked to tumorigenesis in mammary tissue. However, the expression of canine BRCA2 in mammary tumors is unclear.

**Results:**

BRCA2 mRNA levels were compared between seven mammary gland samples and seventeen mammary tumor samples isolated from dogs. The expression level of canine BRCA2 in mammary tumor samples was lower than levels in mammary gland samples. We attempted to identify why the BRCA2 expression level was decreased in mammary tumor samples by promoter sequencing analysis; however, we did not find any mutations in the canine *BRCA2* promoter that altered *BRCA2* transcription levels. We did detect two types of BRCA2 splice variants in 8 mammary tumor samples. One of the variants induced a frame-shift mutation that could lead to nonsense-mediated mRNA decay, a ubiquitous cellular mechanism that eliminates mRNA containing a premature termination codon.

**Conclusions:**

Reduced expression of canine BRCA2 mRNA in mammary tumor samples is a possible mechanism to explain mammary tumor development in dogs. One possible reason for reduced BRCA2 mRNA levels in these tumor samples was nonsense-mediated mRNA decay, not mutations in the *BRCA2* promoter region. While it remains unclear why canine BRCA2 expression levels are reduced in mammary tumor samples, this study found that the expression level of BRCA2 was associated with canine mammary tumorigenesis.

**Electronic supplementary material:**

The online version of this article (doi:10.1186/s12917-015-0483-9) contains supplementary material, which is available to authorized users.

## Background

Mammary tumors are the most common tumor type in women, and they are also the most prevalent tumors in intact female dogs [1–4]. Mammary tumors constitute about half of all tumors in female dogs, and approximately half of canine mammary tumors are malignant [5, 6]. In humans, inheritable breast cancers have been linked with mutations in the breast cancer susceptibility gene, breast cancer 2 early onset (*BRCA2*), and the lifetime risk of developing breast cancer is high (81–88 %) in females carrying a *BRCA2* mutation [7, 8]. A recent study suggested that the canine *BRCA2* locus could also be associated with benign and malignant mammary tumors. This study was based on a single nucleotide polymorphism analysis of an intronic marker [9]. In support of this notion, we previously showed loss of heterozygosity, a mechanism of *BRCA2* inactivation, in a canine mammary tumor sample. Furthermore, canine *BRCA2* missense mutations, including some that affect BRCA2 function, were also reported in canine mammary tumors [10–13].

The BRCA2 protein is involved in homologous recombination repair via its interaction with RAD51 recombinase, and this function suppresses tumorigenesis. BRCA2 expression levels are important for preventing tumorigenesis, because cells with decreased expression of BRCA2 have heightened sensitivity to agents that induce DNA damage, such as DNA cross-linking agents and ionizing radiation [14, 15]. In other words, the reduced BRCA2 expression leads to tumor formation. One of the most likely reasons why BRCA2 mRNA levels are reduced is a mutation in its promoter region. In humans, recent study showed that single nucleotide polymorphisms around the *BRCA2* gene affected the expression levels of human BRCA2 mRNA and increased breast cancer risk [16, 17].

Another pathway that may reduce BRCA2 expression is nonsense-mediated mRNA decay (NMD), a ubiquitous cellular mechanism that eliminates mRNAs containing premature termination codons (PTCs) to prevent the synthesis of potentially harmful truncated proteins [18]. Nonsense mutations and splice variants inducing frame-shift mutations downstream of BRCA2 lead to PTC. Some PTC-induced mutations in human BRCA2 were associated with NMD [19].

However, in canine mammary tumors, little is known about BRCA2 expression. In this study, we found reduced BRCA2 mRNA expression levels in canine mammary tumors. To determine the reason why BRCA2 expression was decreased in canine mammary tumors, we performed a mutation analysis of the canine *BRCA2* promoter region and splice variants, the transcript of which may lead to PTC and NMD.

## Results

### Reduced BRCA2 expression levels in canine mammary tumors

**Fig. 1:**
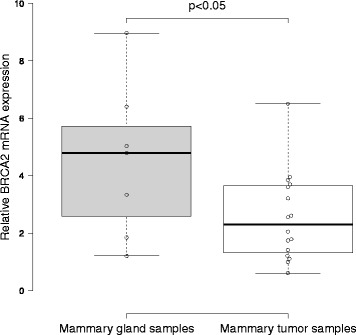
mRNA expression levels of canine BRCA2 in normal mammary gland and mammary tumor samples. Total RNA was prepared from seven normal mammary gland and seventeen mammary tumor samples. The relative expression levels of canine BRCA2 were analyzed by quantitative real-time PCR using canine RPS18 as an internal control. The expression level of canine BRCA2 mRNA in mammary tumor samples was significantly lower than that in mammary gland samples (p < 0.05, Mann–Whitney *U* test)

The expression levels of tumor suppressor genes such as BRCA2 are important for preventing tumorigenesis in mammary cells. Thus, we hypothesized that tumorigenesis may be associated with low levels of BRCA2 expression. We first tested the expression levels of canine BRCA2 in mammary gland and mammary tumor samples by qRT-PCR. We designed primers for exons 26 and 27, the last two exons of canine BRCA2. As expected, the BRCA2 expression level in canine mammary tumors was significantly lower than the level in normal mammary gland tissues (Fig. 1).

### Mutation and promoter activity analyses in the canine *BRCA2* promoter region in mammary tumor samples

**Fig. 2:**
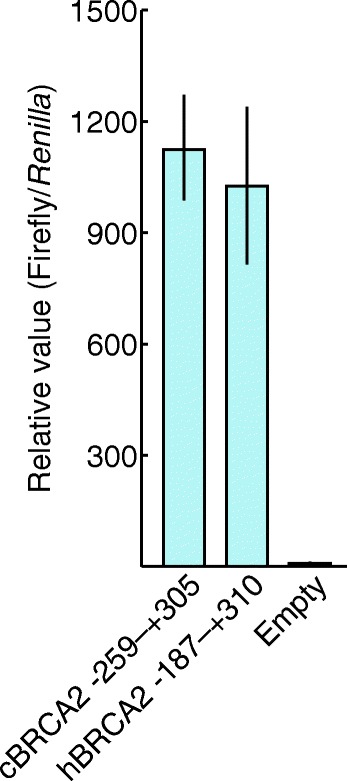
Determination of the canine *BRCA2* promoter region. To determine the sequence of canine *BRCA2* promoter region, the region corresponding to the human *BRCA2* promoter was cloned into the pGL4 vector. The transfection efficiency was normalized with the co-transfection and measurement of *Renilla* luciferase activity. Results shown represent mean ± SD of triplicate experiments

One possible reason for reduced canine BRCA2 expression is a mutation in the canine *BRCA2* promoter region. However, the promoter region of canine *BRCA2* was never studied. Therefore, we first determined the canine *BRCA2* promoter region. In humans, the genomic region from-187 bp to +310 bp at the BRCA2 locus was reported as *BRCA2* promoter [20]. The putative canine *BRCA2* promoter region (-259 bp to +305 bp), which included the region corresponding to the human *BRCA2* promoter, was first cloned into the non-promoter pGL4 vector, encoding firefly luciferase (Additional file 1: Figure S1). Luciferase activity in this putative canine *BRCA2* region was comparable to that at the human *BRCA2* promoter (Fig. 2). Thus, we concluded that this putative promoter of canine *BRCA2* functioned as the true canine *BRCA2* promoter.

**Fig. 3:**
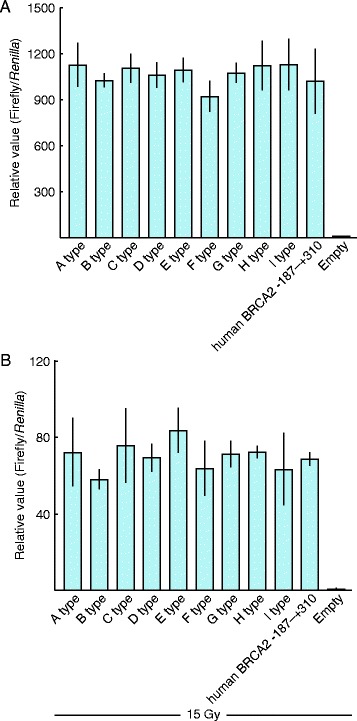
The effect canine *BRCA2* promoter region variation on promoter activity. Canine *BRCA2* promoter regions, containing each variation, were cloned into the pGal4 vector. Promoter activity was analyzed by luciferase assay without or with DNA damage by 15 Gy irradiation (**a** and **b**). The promoter activity in all allele types was similar. There were no significant differences among the allele types (one-way ANOVA). Results shown represent mean ± SD of triplicate experiments

We next performed a mutation analysis of the *BRCA2* promoter region in the canine mammary tumor samples. We sequenced the DNA within this region and identified 9 allele types in canine mammary tumor samples (Table 1; genotypes of each tumor sample are shown in Additional file 2: Table S1). These variations were located in the putative cis-element, which corresponds to the site necessary for human BRCA2 expression (Additional file 1 Figure S1). We next investigated the effects of these variations on canine *BRCA2* promoter activity using a luciferase reporter assay. However, none of these variations affected the activity of the canine *BRCA2* promoter with or without DNA damage by X-ray irradiation (Fig. 3 a and b).

**Table 1:** Promoter region allele types in mammary tumor samples

Allele type	Base sequence								Allele frequency
	–162	–156	–118	–117	–100	–95	–37	+9	
A type	–	–	–	–	–	–	–	–	11/34
B type	–	C	–	–	–	–	–	–	5/34
C type	–	–	–	–	–	–	T	–	3/34
D type	–	C	–	–	–	–	T	–	4/34
E type	–	C	delCGCCCCGC	–	–	–	–	–	1/34
F type	–	–	delCGCCCCGC	–	–	–	T	–	3/34
G type	A	–	delCGCCCCGC	–	–	–	T	–	4/34
H type	A	–	–	–	delTGCCCCC	–	–	–	1/34
I type	–	–	–	delGCCCCGT	–	delCCTGCCCCCTGCCC	T	delCGGCGG	2/34

### Detection of splice variants

**Fig. 4:**
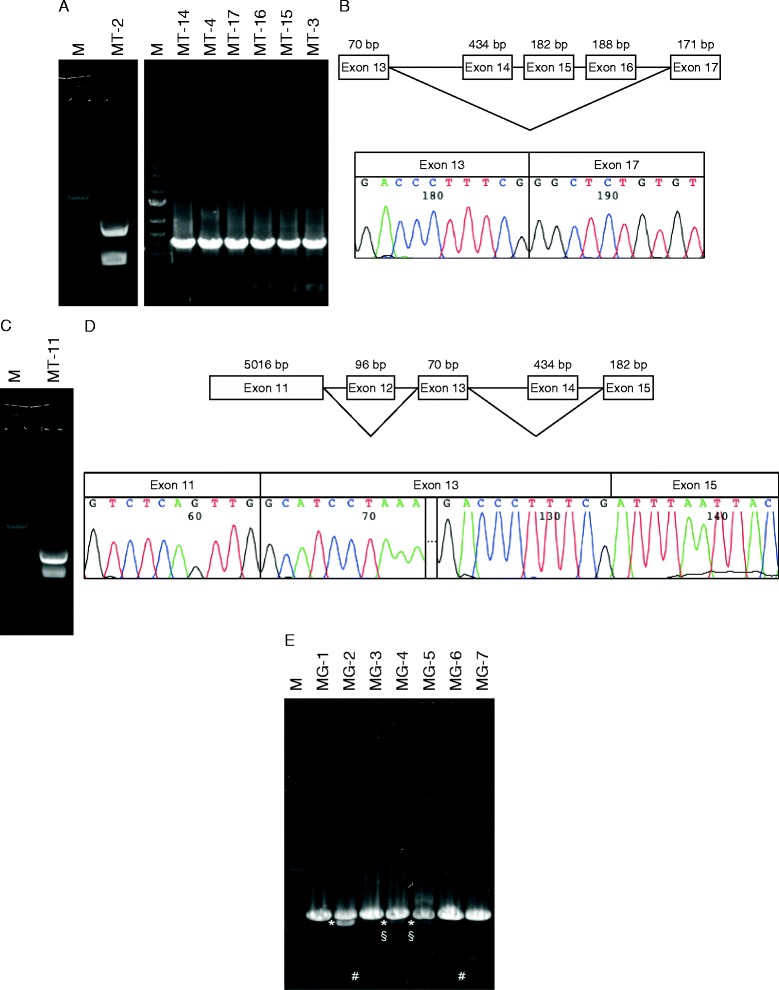
Detecting splice variants in mammary tumor and mammary gland samples. **a** Splice variants missing exons 14-16 were detected in mammary tumor samples. The left panel shows the PCR products detected by 1 % agarose gel electrophoresis, and the right panel shows the DNA sequence chromatogram. **b** Splice variant missing exons 12 and 14 were detected in mammary tumor samples. The left panel shows the PCR products detected by 1 % agarose gel electrophoresis, and the right panel shows the DNA sequence chromatogram. **c** Splice variants detected in mammary gland samples. Symbols are as follows: *, splice variant missing exon 12; §, splice variant missing exon 14; #, splice variant missing exons 13-16

Another reason for decreased mRNA expression is NMD, a ubiquitous cellular mechanism that eliminates PTC containing mRNAs to prevent the synthesis of truncated proteins that may be detrimental to cells [18]. One of the causes of PTC is a frame shift mutation induced by a splice variant. We next attempted to detect BRCA2 splice variants, which may contain frame-shift mutations, in mammary tumor samples using a modified method that we reported previously [21]. In our previous study, we established primer sets to detect splice variants of canine BRCA2. In this study, we chose a primer set that amplified the region encoding exons 11 to 18. This region was chosen because splice variants have been detected for a homologous region in human *BRCA2*, and we speculated that canine mammary tumors might show a similar result [22, 23]. Indeed, we detected two splice variants: one lacking exons 14–16, and the other lacking exons 12 and 14. The variant lacking exons 12 and 14 showed a frame-shift mutation and PTC in 1 out of 17 canine mammary tumor samples (Fig. 4). In two mammary tumor samples (MT-2 and MT-11), the expression level of the splice variant was comparable to that of the wild-type splicing form. Surprisingly, even in the PCR products from normal canine mammary gland tissues, we detected a faint signal of the splice variants (these transcripts lacked exon 12, 14, or 13–16); however, the wild-type splicing form accounted for the majority of mRNA transcripts.

We also sequenced exon-intron boundary region of the 8 mammary tumor samples where splice variants were detected, as mutations at these boundaries induce splice variants. However, there were no mutations in these regions in any of the 8 tumor samples analyzed.

## Discussion

Reduced BRCA2 expression disrupts homologous DNA recombination repair mechanisms, leading to tumor formation [14, 15]. In this study, we compared the expression levels of canine BRCA2 in mammary gland tissue and mammary tumor samples. We found that BRCA2 expression was significantly reduced in mammary tumor samples compared to mammary gland samples. These results suggested that the low expression of canine BRCA2 may be related to mammary tumor development in dogs.

We further investigated possible reasons why canine BRCA2 expression was reduced in tumor samples: mutations in the *BRCA2* promoter region and non-sense mRNA decay (NMD) induced by PTCs that can form in splice variants. To do this, we first identified and sequenced the canine *BRCA2* promoter region. We found that some cis-elements in human *BRCA2* promoter were not conserved. This suggests that regulatory mechanisms controlling *BRCA2* transcription are different in dogs or perhaps that there are unknown cis-elements in the canine and human *BRCA2* promoters [24, 25]. Regardless the differences in cis-elements between the canine and human *BRCA2* promoter region, we found that the promoter activity of the canine *BRCA2* promoter was comparable with human *BRCA2* promoter. A *BRCA2* promoter sequence analysis of mammary tumors revealed nine promoter allele types. Interestingly, these variations were located near the corresponding site of a human BRCA2 cis-element; however, none of the alleles disrupted canine *BRCA2* promoter activity, including in cases with DNA damage by X-ray irradiation. These results led us to conclude that the nine alleles harbored neutral mutations and that they were not associated with tumorigenesis.

We also explored whether the NMD system induced by PTCs could account for reductions in BRCA2 levels. One way to form PTCs is through splice variants. Detection of splice variants is more efficient than analyzing the entire BRCA2 sequence because BRCA2 is a large gene product (more than 11 kbp). We detected splice variants in 8 out of 17 canine mammary tumor samples and 5 out of 7 normal mammary gland samples. In two of the mammary gland samples, the expression level of the splice variants was comparable to that of the wild-type splicing form. Because, one of the splice variants lacking exons 12 and 14 induced frame-shift and PTC, it is possible that canine BRCA2 mRNA levels were reduced by these splice variants inducing PTC and the NMD system. Although this splice variant would not affect the mRNA levels of canine BRCA2, the transcripts would not have nuclear localization signals located at the C-terminal and would not function in the nuclei [26, 27]. Because the most important function of BRCA2 is DNA repair in the nuclei, another possibility is that the splice variant lacking exons 14–16 is associated with mammary tumors. The splice variant lacking exons 14–16 does not induce PTC. Its transcript lacks the front part of helical domain, where BRCA2 interacts with DSS1, a stabilizer [28, 29] . Thus, the transcript from the splice variant lacking exons 14–16 would be unstable and could be associated with mammary tumors.

One of variant transcripts found in mammary gland samples was missing exon 12; this variant was also reported in human BRCA2 [22]. Exon 12 encodes a 96 bp region of both canine and human *BRCA2*; therefore, this particular splice variant does not induce a frame-shift mutation. Human *BRCA2* transcripts missing exon 12 did not have any effect on DNA repair activity. Thus, it was suggested that canine *BRCA2* transcripts missing exon 12 did not affect its function and expression levels. The expression levels of the other two splice variants, which were missing exon 14 or exons 13–16, were very low compared to that of wild-type transcripts. We assumed that the low expression level of these splice variants did not affect BRCA2 mRNA expression. We also tried to determine the presence of mutations in the exon-intron boundary regions of the *BRCA2* transcripts, which is the most likely reason for the existence of splice variants. However, we did not detect any mutations in these regions. This result suggested that the splicing machinery might not function properly, resulting in BRCA2 splice variants.

Other reasons for the reduction in canine BRCA2 mRNA levels are still unclear. One possible explanation may be unregulated miRNA expression. Recently, the discovery of some miRNAs regulating BRCA2 expression levels were linked to breast cancer development in humans [30]. Therefore, it is possible that the expression of miRNAs could decrease canine BRCA2 expression at the mRNA level; however, it is unknown whether the same miRNA mechanism regulating human BRCA2 expression is functional in canines. Further studies are needed to address this issue.

Further studies are needed to reveal the correlation between canine BRCA2 transcript and protein levels in mammary tumor samples. Unfortunately, in this study, we collected mammary tumor samples soaked in RNAlater solution; therefore, we could only test BRCA2 mRNA levels and could not determine BRCA2 protein levels by western blotting or immunohistochemistry. In future studies, mammary tumor samples should be collected in two batches: one for RNA extraction and the other for western blotting or immunohistochemistry.

## Conclusions

In this study, we showed that canine *BRCA2* mRNA transcript levels were significantly reduced in mammary tumor samples compared to normal mammary gland tissue. One of the reasons for reduced BRCA2 expression was nonsense-mediated mRNA decay (NMD), a ubiquitous cellular mechanism that eliminates premature termination codon that can be found in splice variants. Our results suggested that low BRCA2 expression in canine mammary tumors is a possible mechanism that leads to tumorigenesis. However, we still need to elucidate the mechanisms that regulate BRCA2 expression levels, and how low BRCA2 expression leads to the development of canine mammary tumors.

## Methods

### Mammary gland and tumor samples

Seven mammary gland samples and 17 mammary tumor samples were obtained from the veterinary hospitals of Iwate University and Kitasato University. These samples were stored at –20 °C in RNAlater solution (Life Technologies) until processed for DNA or RNA extraction. All experimental procedures were approved by and conducted in accordance with the Guidelines for Institutional Laboratory Animal Care and Use of the School of Veterinary Medicine at Kitasato University, Japan (Approval Number: 11–065).

### Quantitative real-time PCR

RNA extraction and cDNA synthesis were performed as previously described [21]. Quantitative real-time polymerase chain reaction (qRT-PCR) was carried out on StepOnePlus Real-Time PCR systems (Life Technologies) using KAPA SYBR FAST qPCR Kit (Kapa Biosystems) and 200 nM of each primer (Additional file 3: Table S2). The PCR cycling conditions were as follows: 95 °C for 20 s followed by 40 PCR cycles of 95 °C for 3 s and 60 °C for 30 s. Melting curves were generated at the end of each real-time PCR run to ensure that a single specific product was amplified. Each sample was run in triplicate. The housekeeping gene canine RPS18 was used as internal references for normalization.

### Genomic PCR and Sequence analysis

DNA extraction was performed as previously described [21]. To amplify the canine and human *BRCA2* promoter region, each reaction mixture contained 10-50 ng of genomic DNA as a template, each forward and reverse primer (Additional file 3: Table S2) at 300 nM, 200 μM dNTPs, 0.02 U of KOD FX DNA polymerase (Toyobo, Japan), and 1x PCR buffer, which was supplied with the enzyme, in a total volume of 10 μl. PCR conditions included one cycle of 2 min at 94 °C, followed by 35 cycles of 10 s at 98 °C, 15 s at the appropriate temperature for primer annealing, 1 min at 68 °C, and a final extension step of 7 min at 68 °C. The PCR products were treated with shrimp alkaline phosphatase (Affymetrix, Santa Clara, CA) and Exonuclease I (New England BioLabs, Beverley, MA) before sequencing. Direct DNA sequencing was performed at least twice for each amplicon. For some PCR products with heterozygous deletion mutations, direct sequencing could not determine the DNA base sequence; instead, PCR products were cloned into a pMD20 vector (TaKaRa, Japan) and at least five independent clones were sequenced to determine the heterozygous insertion/deletion mutations.

### Luciferase assay

For analysis of promoter activity using luciferase, the canine and human *BRCA2* promoter regions were cloned into pGL4 plasmids (Promega, U.S.A.). Approximately 2 × 10^5^ HeLa cells were transiently co-transfected with 100 ng pGAL4 and 1 ng pRL-TK (Promega) using FuGENE HD transfection reagent (Roche Diagnostics, Basel, Switzerland). Forty-eight hours after transfection, the cells were lysed in passive lysis buffer (Promega). Firefly and Renilla luciferase activity was measured using the Dual-Luciferase Reporter Assay Kit (Promega) according to the manufacturer’s instructions. The transfection efficiency was normalized by measuring the *Renilla* luciferase activity.

To test the DNA damage caused by X-ray irradiation, at 3 h after transfection the cells were irradiated with 15 Gy and the irradiated cells were harvested 15 h later.

### Detection of splice variants

To detect splice variants and determine the DNA sequences of exons 11–26, including the exon-intron boundary region in canine *BRCA2*, the target regions were amplified and sequenced as described previously [21]. However, the DNA samples derived from mammary tumors did not amplify in the first PCR reaction, thus in these samples, nested PCR was performed using a second primer set (Additional file 3: Table S2). The nucleotide sequence of these PCR products was determined by direct sequencing.

### Statistical analysis

To compare the canine BRCA2 mRNA expression level between mammary grand and mammary tumor samples, the Mann–Whitney *U*-test was used. For comparison of canine BRCA2 promoter activity among variants, one-way Analysis of Variance (ANOVA) tests were used.

## Additional files

Additional file 1: Figure S1. Alignment of the canine and human *BRCA2* promoter regions. To identify the canine *BRCA2* promoter region, the putative promoter region was aligned with the human *BRCA2* promoter. Boxes indicate important cis-elements in the human *BRCA2* promoter region. Arrowheads indicate the sites where we identified variations in mammary tumor samples. Additional file 2: Table S1. Genotype of canine BRCA2 in mammary tumor samples. Additional file 3: Table S2. Primer sets used in this study.
