# Supplementary material for: Reduced canine BRCA2 expression levels in mammary gland tumors
Source: BMC Vet Res. 2015 Jul 23;11:159. doi: 10.1186/s12917-015-0483-9 (PMC4512014; doi:10.1186/s12917-015-0483-9)
Supplement: Additional file 1: Figure S1. — Alignment of the canine and human BRCA2 promoter regions. To identify the canine BRCA2 promoter region, the putative promoter region was aligned with the human BRCA2 promoter. Boxes indicate important cis-elements in the human BRCA2 promoter region. Arrowheads indicate the sites where we identified variations in mammary tumor samples. [file 12917_2015_483_MOESM1_ESM.pdf]

```
hBRCA2 -187 -----------------------TCAGCGAGAAGAGAACACACACTCCAGCTCCCGCTTT
cBRCA2 -259 CGCCGCGTCGAGCTGCGAAGGGCTCAGGGAGAGGAGAGCGAGCACCCCGACTCCCTCTTC
                                   **** **** **** *   *** **  ***** ***

hBRCA2 -150 ATTCGGTCAGATACTGACGGTTGGGATGCCTGACAAGGAATTTCCTTTCGCCACACTGAG
cBRCA2 -199 ACACGGTTCCAGGCCGACCGCCCAGCAGCCGAACGAGGACTCGGCTTTCGCCACACGGAC
            *  ****   *  * *** *    *  ***  ** **** *   ************ **

hBRCA2  -90 AAAT-----------------------ACCCGCAGCGGCCCACCCAGG-CCTGACTTCCG
cBRCA2 -139 GCGCCCGCCGCCCGCCGCCCGCTCCCCGCCCCCTGCCCCCTGCCCAGAACCCGACTTCCG
                                        *** * **  **  *****  ** ********


hBRCA2  -54 GGTGGTGCGTGTGCTGCGTGTCGCGTCACG---------GCGTCACGTGGCCAGCGCGGG
cBRCA2  -79 GGAGGCGCGCACGCTGCGTGTCGCGTCATGCGTCACTGCGTGCCGCGTCGGCGGAGCGGG
            ** ** ***   **************** *         * * * *** * * * *****

hBRCA2   -3 CTTGTGGCGCGAGCTTCTGAAACTAGG-CGGCAGAGGCGGAGCCGCTGTGGCACTGCTGC
cBRCA2  -19 TTTTTGGCGCGAGGATCTGAAAGAAGGTCGGCGGAGGCGGAG-----GCGGAGCTGCTGG
              ** *********  *******  *** **** *********     * **  ******

hBRCA2  +57 GCCTCTGCTGCGCCTCGGGTGTCTTTTGCGGCGGTGGGTCGCCGCCGGGAGAAGCGTGAG
cBRCA2  +37 GGCTTGGC---GCTCTGGAAGTCGTCC-CAGCCGCGGGTCGCCGAGGAAAGGAGCCTGCG
            * **  **   **   **  *** *   * ** * *********  *  ** *** ** *

hBRCA2 +117 GGGACAGATTTGTGACCGGCGCG-------GTTTTTGTCAGCTTACTCCGGCCAAAAAAG
cBRCA2  +93 GGT-CAGCTTTCTGGCCGAAGTGCCGGCGCGAATTTGTTAGCCGTCTCCGGCCAAAAA-G
            **  *** *** ** ***  * *       *  ***** ***   ************* *

hBRCA2 +170 AACTGCACCTCTGGAGCGGGTTAGTGGTGGTGGTAGTGGGTTGGGACGAGCGCGTCTTCC
cBRCA2 +151 AGCGGCACCTCGGAAGGCGGTTAGTGTTGGCTG-GGTGGAGACGGACGGGCCTGTCTTCC
            * * ******* * **  ******** ***  *  ****    ***** **  *******

hBRCA2 +230 GCAGTCCCAGTCCAGCGTGGCGGGGGAGCGCCTCACGCCCCGGGTCGCTGCCGCGGCTTC
cBRCA2 +210 GCGGCGTGAGGCG---GCGGGGGCGGCGCCTCTTCTTCACGGGGCGAGGGCCGGGGCCTC
            ** *    ** *    * ** ** ** **  **    * * ***     **** *** **

hBRCA2 +290 TTGCCCTTTG-TCTCTGCCAA------------------
cBRCA2 +267 CTGCCCCCACGGCCTCTGCAGCCTCTCACCCACCGGGAG
             *****    *  ******
```

Supplementary Figure 1 - Alignment of the canine and human *BRCA2* promoter regions. To identify the canine *BRCA2* promoter region, the putative promoter region was aligned with the human *BRCA2* promoter. Boxes indicate important cis-elements in the human *BRCA2* promoter region. Arrowheads indicate the sites where we identified variations in mammary tumor samples.
